# Ten-Year Survival in 75-Year-Old Men and Women: Predictive Ability of Total Cholesterol, HDL-C, and LDL-C

**DOI:** 10.1155/2009/158425

**Published:** 2009-04-27

**Authors:** Göran Nilsson, John Öhrvik, Ingemar Lönnberg, Pär Hedberg

**Affiliations:** ^1^Centre of Clinical Research, Västerås Central Hospital, Uppsala University, SF-751 05 Uppsala, Sweden; ^2^Department of Medicine, Cardiology Research Unit, Karolinska Institute, SE-17176, Stockholm, Sweden; ^3^Department of Cardiology, Central Hospital, SE-72189 Västerås, Sweden; ^4^Department of Clinical Physiology, Central Hospital, SE-72189 Västerås, Sweden

## Abstract

*Objective*. The purpose of this study was to investigate prognostic impact of cholesterol and its subfractions among 75-year-old people from the general population. *Methods and Results*. The study comprised a random sample (222 women and 210 men) from the general population (participation rate 70%). During 10-year follow-up, 19% of women and 35% of men experienced a major cardiovascular event (MCVE). The all-cause mortality was 29% for women and 47% for men. After adjustment for cardiovascular risk factors, a low level of high-density lipoprotein cholesterol (HDL-C) was significantly associated with MCVE (*P* = .006) and mortality (*P* = .011) in men but not in women. The prognostic sex disparity was nearly significant (*P* = .051 for MCVE and .067 for mortality). The associations of adjusted HDL-C to MCVE and mortality were unchanged after excluding individuals with prevalent stroke or MI. Total cholesterol and low-density lipoprotein cholesterol (LDL-C) were not significantly related to prognosis in either sex. *Main Conclusions*. HDL-C was associated with dismal prognosis in men but not in women. Elderly men with HDL-C <40 mg/dL deserve particular attention for cardiovascular prevention.

## 1. Introduction

In middle-aged women and men there is a strong and graded association between total cholesterol and low-density lipoprotein cholesterol (LDL-C) concentrations and cardiovascular events as well as total mortality [1–4]. On the contrary, in middle-aged people low levels of high-density lipoprotein cholesterol (HDL-C) have been shown to be an independent risk factor of atherosclerotic disease [4–7]. However, in elderly people, reports on whether survival and atherosclerotic disease relates to the levels of cholesterol and its fractions are conflicting [4, 8–11]. In particular, reports on the relationship between survival and the serum levels of total cholesterol, LDL-C, and HDL-C are scanty in women above 75.

Each age class represents survivors from younger age classes. Therefore, risk indicators may not be the same in different age groups. Thus, persons suffering adverse effects of abnormal levels of cholesterol and its subfractions may have died before the age of 75, leaving room for new risk factor patterns to emerge. Among the oldest people the mortality increases rapidly, and it is therefore reasonable to assume a rapidly changing risk factor pattern. In risk factor analyses, all elderly people above a certain age, for instance 65 or 70, are usually evaluated as a homogenous group. Such a practice may hide important details. Furthermore, the risk factor patterns of a certain age class may change over decades. The risk factor patterns of 75-year old people may nowadays be different from that found some decades ago.

 The purpose of this study was to investigate the relations between the serum levels of total cholesterol and its subfractions and long-term prognosis in a cohort of 75-year-old men and women.

## 2. Methods

### 2.1. Study Population

The study population has previously been described elsewhere [12]. In 1997 there were 1100 inhabitants of the city of Västerås, born in 1922, making them 75 years of age. A random sample of 618 of these individuals was invited to participate in a cardiovascular health survey. The city of Västerås, with a total population of 130 000, is situated in the central part of Sweden, and the population is representative of Sweden from a socioeconomic point of view. The invitation was accepted by 433 subjects (223 women and 210 men) corresponding to a participation rate of 70%. The reasons for nonparticipation were that the individual could not be reached (*n* = 29), the person had died before the examination (*n* = 2), language difficulties or logistical problems (*n* = 26), locomotive impairment (*n* = 28), unwillingness because of disease under treatment (*n* = 54), or unknown (*n* = 46). The blood sample from one woman was missing, and consequently the examined cohort included 222 women and 210 men. The study was approved by the research ethics committee of Uppsala University, Sweden.

### 2.2. Baseline Examination

Blood samples were collected in the morning with the subjects in a fasting state. Total serum cholesterol and HDL-C were determined enzymatically using an automated analyzer system (Hitachi 717, Boehringer Mannheim). Non-HDL-C was defined as total cholesterol minus HDL-C. LDL-C was calculated using the Friedewald formula (13). This formula does not permit computing LDL-C at triglyceride levels >400 mg/dL. Consequently LDL-C values were missing in 8 persons. The coefficient of variation for total cholesterol was 1.5% at 124 mg/dL and 1.6% at 297 mg/dL; for HDL-C 2.7% at 34 mg/dL and 2.7% at 93 mg/dL.

 Blood pressure was measured with a mercury sphygmomanometer with the subjects in a supine position and having relaxed for five minutes. Diagnosis of previous myocardial infarction, stroke, and diabetes was based on self-reported history of disease verified by medical records. Hypertension was defined as self-reported physician-diagnosed high blood pressure in combination with regular antihypertensive treatment.

### 2.3. Follow-Up

Major cardiovascular event (MCVE), defined as hospitalization or death caused by myocardial infarction, stroke, or ruptured abdominal aortic aneurysm, was selected as the primary end-point. The secondary end-point was all-cause mortality. Follow-up information on MCVE was based on the Swedish Hospital Discharge register and the Cause of Death register (with available data up until December 31, 2006). The tenth revision of the International Statistical Classification of Diseases (ICD) was used to identify myocardial infarction (I21–I22), stroke (I61–I66), and ruptured abdominal aortic aneurysm (I71.3). Information on all-cause death was based on the Swedish Population register (with available data up until December 31, 2007). The registers were linked to the individuals by the unique personal identification number of all citizens in Sweden. The participants were followed from the index examination in 1997 until they reach an end-point or December 31, 2006 (MCVE) or December 31, 2007 (all-cause mortality) at which time they were censored.

In order to achieve information from the Hospital Discharge register, a renewed written informed consent was required from the participants still alive during 2007. Because of difficulties in reaching some participants and refusal from others, 38 persons were lost to follow-up in this register, leaving 394 individuals to be analyzed regarding the MCVE. No participants were lost to follow-up concerning all-cause mortality.

### 2.4. Statistics

Continuous variables were summarized by median and interquartile range and categorical variables by counts and proportions. The Wilcoxon Mann-Whitney rank sum test was used to compare groups for continuous variables. Categorical variables were compared using a two-sided Fisher's exact test.

The relations of cholesterol and its subfractions to outcome were investigated by the use of Cox proportional hazards regression models in sex-stratified univariable analyses. The assumption of proportionality of hazards was assessed by including time-dependent covariates in the models. The relations of HDL-C to MCVE and all-cause mortality were evaluated by multivariable Cox regression models adjusting for potential risk factors (body mass index, smoking, non-HDL-C, triglycerides, diabetes, hypertension, and previous myocardial infarction). For adequate control of confounders, covariates were retained in the models regardless of their statistical significance. To investigate the sex difference in the strength of association between HDL-C and outcome, we besides HDL-C included sex and the interaction between sex and HDL-C in a multivariable Cox regression model. To detect potential collinearity that could disturb our analyses, forward and backward stepwise analyses were performed. The inclusion and removal probability limits were set at 0.05 and 0.10.

Survival curves were generated by means of Kaplan-Meier estimates, and differences in survival were compared by the log-rank test. All tests were two tailed and a *P*-value <.05 was considered statistically significant. The SPSS version 14.0 (SPSS Inc., Chicago, Ill) was used for all analyses.

## 3. Results

Baseline characteristics of the examined cohort are shown in Table 1. There was a notable sex disparity concerning serum levels of HDL-C and total cholesterol as well as concerning prevalence of previous myocardial infarction.

During a median follow-up period of 9.5 years, 38 (19%) women and 68 (35%) men experienced an MCVE (number of MCVE per year per 100 persons at risk 2.3 for women and 4.9 for men, *P* < .001). Specifically, the registered events were nonfatal and fatal myocardial infarction (*n* = 38 and *n* = 17, resp.), nonfatal and fatal stroke (*n* = 34 and *n* = 10, resp.), and nonfatal and fatal aortic rupture (*n* = 1 and *n* = 6, resp.). Concerning all-cause mortality, the median follow-up was 10.5 years during which 65 (29%) women and 98 (47%) men died (number of death per year per 100 persons at risk 3.1 for women and 5.5 for men, *P* < .001).

Serum levels of HDL-C were significantly related to both MCVE and all-cause mortality in men but not in women (Table 2). These associations did not substantially change after adjustment for the potentially confounding risk factors. In both forward and backward stepwise multivariable Cox regression analyses, HDL-C (together with smoking and hypertension) was retained as a significant predictor of both MCVE and all-cause mortality in men. The corresponding stepwise analyses in women expelled HDL-C from the models for both MCVE and all-cause mortality. Smoking, diabetes, and previous myocardial infarction were retained as independent predictors of MCVE in the stepwise models in women, whereas smoking and previous myocardial infarction were retained as independent predictors of all-cause mortality. Including sex and the interaction between sex and HDL-C and adjusting for potentially confounding risk factors in a multivariable Cox regression analysis showed a borderline significant sex difference in the strength of the association between HDL-C and time to MCVE (*P* = .051) and to all-cause mortality (*P* = .067).

When excluding individuals with prevalent stroke or myocardial infarction at baseline, the association of adjusted HDL-C to MCVE was unchanged in both men and women, whereas the association to all-cause mortality was slightly strengthened. In men the resulting hazard ratio of adjusted HDL-C to all-cause mortality was 0.68 (95% CI 0.54–0.87, *P* = .002) and in women .88 (95% CI 0.72–1.06, *P* = .18). Excluding the 15 individuals on lipid lowering therapy did not result in any substantial changes in the results of the Cox regression analyses.

The National Cholesterol Education Program Adult Treatment Panel III (NCEP-ATP III) has recommended HDL-C levels of <40 mg/dL for men and <50 mg/dL for women as one of the components to define the metabolic syndrome [13]. Some characteristics of the 75-year-old men with HDL-C levels <40 mg/dL or ≥40 mg/dL are shown in Table 3. Men with low HDL-C had significantly higher prevalence of diabetes and hypertension as well as significantly higher levels of serum triglycerides than those with high HDL-C. The difference in plasma glucose was nearly significant.

**Figure 1:**
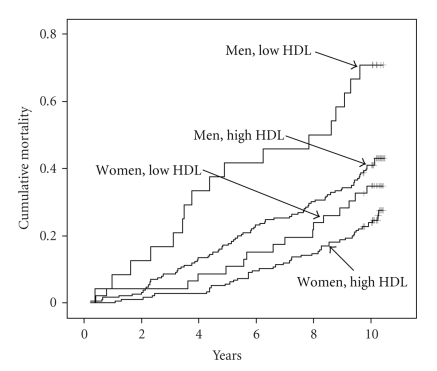
Kaplan-Meier curve illustrating all-cause mortality according to cutoff levels of HDLC as recommended by the National Cholesterol Education Program—Adult Treatment Panel III (men: low HDL <40 mg dL, high HDL = 40 mg dL; women: low HDL <50 mg dL, high HDL = 50 mg dL). Men (women) at risk at 2,4,6, and 8 years was 197 (218), 172 (213), 152 (198), and 138 (185).

Figure 1 shows Kaplan-Meier curves for all-cause mortality of men and women with low and high HDL-C according to the NCEP-ATP III recommended cutoff levels. Twenty-four men and 46 women were thereby classified by these limits as having low HDL-C. The difference in survival between the low and high HDL-C levels was significant for men (log-rank statistics = 8.92, *P* = .003) but not for women (log-rank statistics = 2.86, *P* = .091).

## 4. Discussion

### 4.1. HDL-C

The present investigation comprises 70% of all 75-year-old people in a defined geographical area and reasonably reflects a general population of this age. The main finding was that low serum levels of HDL-C were considerably stronger associated with increased cardiovascular morbidity and mortality in men than in women during long-term follow-up in a population-based cohort of 75 year-old people. On the contrary, the levels of total cholesterol, LDL-C and non-HDL-C had no significant impact on the long-term prognosis in either sex.

It is well known that a low serum level of HDL-C is a powerful predictor of increased cardiovascular risk in young and middle-aged people [3, 5, 14–16]. Interestingly, this association was observed even among patients with LDL-C concentration below 70 mg/dL during statin treatment [6].

Similar findings as ours concerning HDL-C, survival, and sex have been reported from a 13-year follow-up study of a Finnish population, 65–74 years old, covering 35% of all residents in a defined geographical area (participation rate 71%) [17] and the Bronx aging study concerning 75–85-year-old men and women [9]. However, a recent meta-analysis found no sex disparity in the negative association between HDL-C and cardiovascular outcome in the elderly [4]. 

Among elderly men, our findings concerning HDL-C are consistent with results from the Whitehall population, mean age 77 [7], and a Swedish study of 785 men, aged 77 [10]. Lower levels of HDL-C have been found to be a discriminator of cardiac events among 60-year-old women in the Nurses' Health Study [18]. 

The median HDL-C level was 16% lower in men than in women, and it might be hypothesized that this difference is related to the sex disparity in prognostic impact of HDL-C demonstrated by us. However, in middle-aged people, the inverse relationship between HDL-C and coronary heart disease extends over a broad range of HDL-C levels [14], including the range found in our elderly cohort. The clinical laboratory report of HDL-C refers to the mass of cholesterol within the HDL-C particles. Given the extraordinary biological diversity of HDL particles, these measurements do not provide much functional information [19]. Therefore, it is difficult to find a biological explanation for the sex disparity in prognostic importance of HDL-C.

### 4.2. Total Cholesterol and LDL-C

Declining total cholesterol levels have been found in people above 65 in the Cardiovascular Health Study [20]. The decline was found to be associated with male gender, advanced age, weight loss, and higher white blood cell count. Weverling-Rijnsburger [21] has reported declining levels of total cholesterol in individuals above 85 years of age.

There is strong evidence of a positive association between cholesterol levels and cardiovascular disease as well as cardiovascular death in young and middle-aged people [1–3, 22–25]. Cardiovascular disease is the leading cause of death among the elderly but the importance of hypercholesterolemia as a risk factor in persons above 70 remains controversial. Most studies have noted that the risk weakens with advancing age [4, 26]. Some prospective observational studies have reported positive associations between total cholesterol levels and both cardiovascular disease as well as all-cause mortality among the elderly [27, 28], whereas other studies have reported no such associations [7, 29, 30]. In people above 85 years, high cholesterol has even been reported to be associated with increased survival [21].

### 4.3. Clinical Implications

In the present investigation there was no increase in mortality above recommended limits for high total cholesterol, LDL-C [13, 25], and the quotient total cholesterol/HDL-C [31]. This calls into question the value of cholesterol lowering in 75-year-old people. However, present studies suggest that, at least in secondary prevention, the benefit of lowering cholesterol is independent of age, though detailed information concerning people in their eighties and nineties is lacking. In the 4S study [32] there was no difference in treatment benefit in patients aged below and above 60 years. Similarly, the pooled analysis of the Pravastatin Atherosclerosis Intervention Program, PROSPER, suggested that risk reduction, if any, was greater in patients older than 65 years [33], which is also confirmed in large-meta analyses of major clinical studies [34, 35]. However an upper age limit for the benefit of lipid-lowering drugs may be suspected in view of the positive association between high cholesterol and longevity in people above 85 years of age [21]. Empirical data on the possible value of cholesterol lowering drugs above 85 years of age are lacking.

Established atherosclerotic disease is common in people above 75, and asymptomatic individuals in this age group are at high risk of developing symptomatic atherosclerotic disease. Consequently, the absolute risk of known or unknown atherosclerotic diseases as well as death from such diseases is considerable. When used for secondary prevention, statin therapy has been reported to be strongly protective of morbidity and mortality in atherosclerotic cardiovascular disease also in people aged more than 75 years [35, 36]. In primary prevention, the AFCAPS/TexCAPS study, which recruited participants aged 43–73 years, showed benefit from cholesterol lowering drugs in both men and women also in the older participants [37]. The benefit of common cholesterol lowering drugs in the elderly despite weakening of cholesterol as a risk factor rises suspicion of benefits from such drugs mediated by non-LDL-C lowering mechanisms.

### 4.4. Strengths and Limitations

The restriction of our investigation to one age class enables us to leave age out of account as a confounding factor, creating the possibility of a meaningful estimation of the relations between survival and serum levels of cholesterol and its subfractions, despite the relatively small number participants of the study. Furthermore, because of high participation rate, the participants are more representative for the population in a defined geographical area than described in most other studies on this topic. These advantages are, however, obtained at the cost of the difficulties to generalize our findings to people not being 75 years old and to people from other geographical areas. However, it seems likely that our results are applicable to North European and white North American people in their seventies.

In conclusion, low serum levels of HDL-C were significantly associated with increased cardiovascular morbidity and mortality in men but not in women during long-term follow-up in this population-based cohort of 75-year-old people. Elderly men with HDL-C <40 mg/dL should be given attention for cardiovascular prevention. The serum levels of total cholesterol, LDL-C, and non-HDL-C had no significant impact on prognosis. This calls into question the value of cholesterol lowering in 75-year-old people.

## Figures and Tables

**Table 1:** Baseline characteristics of the 75-year-old participants. Values are median (interquartile range) and number (%).

	Men (*n* = 210)	Women (*n* = 222)	*P*-value
Total cholesterol, mg/dL^†^	228 (205–259)	252 (224–276)	<.001
LDL-C, mg/dL*^†^	147 (120–173)	159 (132–184)	.001
HDL-C, mg/dL^†^	53 (45–60)	63 (52–75)	<.001
Non-HDL-C, mg/dL^†^	176 (152–206)	187 (156–216)	.010
Total cholesterol/HDL-C ratio	4.3 (3.7–5.2)	3.9 (3.2–5.0)	.003
Triglycerides, mg/dL^†^	134 (98–172)	128 (98–181)	.92
Body mass index, kg/m^2^	25.2 (23.6–26.9)	26.0 (23.4–28.9)	.011
Systolic blood pressure, mmHg*	160 (150–180)	165 (150–190)	.007
Diastolic blood pressure, mmHg*	85 (80–90)	85 (80–90)	.84
Current smoking	34 (16)	19 (9)	.116
Previous myocardial infarction	31 (15)	10 (5)	<.001
Previous stroke	6 (3)	8 (4)	.22
Previously known hypertension	55 (26)	63 (28)	.67
Diabetes	15 (7)	17 (8)	.49
Lipid lowering drugs	8 (4)	7 (3)	.78

*Data on blood pressure and LDL-C were missing in 11 and 8 cases, respectively. ^†^To convert total cholesterol, HDL-C, and LDL-C from mg/dL to mmol/L, divide by 39. To convert triglycerides from mg/dL, to mmol/L divide by 89.

**Table 2:** Hazard ratios for major cardiovascular event defined as hospitalization or death caused by myocardial infarction, stroke, or ruptured aortic aneurysm or all-cause mortality according to serum levels of cholesterol and its subfractions.

	Major cardiovascular event				All-cause mortality			
	Men (*n* = 195)		Women (*n* = 199)		Men (*n* = 210)		Women (*n* = 222)	
	HR (95% CI)*	*P*	HR (95% CI)*	*P*	HR (95% CI)*	*P*	HR (95% CI)*	*P*
Total cholesterol	1.01 (0.96–1.07)	.68	1.00 (0.93–1.07)	.94	0.97 (0.92–1.02)	.29	0.95 (0.90–1.00)	.065
LDL-C	1.02 (0.96–1.09)	.44	1.02 (0.93–1.11)	.72	0.97 (0.92–1.03)	.34	0.99 (0.92–1.06)	.73
Non-HDL-C	1.03 (0.98–1.09)	.25	1.01 (0.95–1.08)	.77	0.99 (0.95–1.04)	.82	0.97 (0.93–1.02)	.20
Total cholesterol/HDL-C ratio	1.19 (1.00–1.40)	.046	1.13 (0.89–1.45)	.32	1.10 (0.95–1.29)	.20	1.07 (0.90–1.29)	.44
HDL-C	0.76 (0.61–0.94)	.011	0.93 (0.78–1.10)	.40	0.78 (0.65–0.94)	.009	0.93 (0.82–1.07)	.32
HDL-C adjusted^†^	0.71 (0.55–0.90)	.006	1.01 (0.83–1.23)	.94	0.76 (0.62–0.94)	.011	0.93 (0.80–1.07)	.31

*Hazard ratios and 95% confidence intervals for every unit increase of the total cholesterol/HDL-C ratio and for every 10 mg/dL increase of the other independent variables. ^†^Adjusted for smoking, non-HDL-C, triglycerides, diabetes, hypertension, previous stroke, and previous myocardial infarction.

**Table 3:** Characteristics of the 75-year-old men with high or low HDL-C. Cutoff level of HDL-C (40 mg/dL) according to recommendation from the National Cholesterol Education Program—Adult Treatment Panel III. Values are median (interquartile range) and number (%).

	HDL-C <40 mg/dL	HDL-C ≥40 mg/dL	*P*-value
	(*n* = 24)	(*n* = 186)	
Total cholesterol, mg/dL	217 (207–246)	232 (205–259)	.33
LDL-C, mg/dL*	147 (127–166)	148 (119–173)	.62
HDL-C, mg/dL	36 (34–38)	55 (49–62)	
Non-HDL-C, mg/dL	179 (172–207)	175 (151–205)	.16
Total cholesterol/HDL-C ratio	6.2 (5.6–6.5)	4.2 (3.5–4.9)	<.001
Triglycerides, mg/dL	214 (142–271)	125 (96–161)	<.001
Body mass index, kg/m^2^	25.4 (23.6–27.4)	25.2 (23.6–26.9)	.32
Systolic blood pressure, mmHg*	170 (142–180)	160 (150–180)	.76
Diastolic blood pressure, mmHg*	90 (80–90)	85 (80–90)	.50
Current smoking	4 (17)	30 (16)	1.00
Previous myocardial infarction	4 (17)	27 (14)	.76
Previously known hypertension	11 (48)	44 (24)	.022
Diabetes	5 (21)	10 (5)	.018
Lipid lowering drugs	0 (0)	8 (4)	

*Data on blood pressure and LDL-C were missing in 11 and 8 cases, respectively.

## References

[1] Stamler J, Wentworth D, Neaton JD (1986). Is relationship between serum cholesterol and risk of premature death from coronary heart disease continuous and graded? Findings in 356,222 primary screenees of the Multiple Risk Factor Intervention Trial (MRFIT). *The Journal of the American Medical Association*.

[2] Stamler J, Daviglus ML, Garside DB, Dyer AR, Greenland P, Neaton JD (2000). Relationship of baseline serum cholesterol levels in 3 large cohorts of younger men to long-term coronary, cardiovascular, and all-cause mortality and to longevity. *The Journal of the American Medical Association*.

[3] De Backer G, Ambrosioni E, Borch-Johnsen K (2003). European guidelines on cardiovascular disease prevention in clinical practice: third joint task force of European and other societies on cardiovascular disease prevention in clinical practice (constituted by representatives of eight societies and by invited experts). *European Journal of Cardiovascular Prevention & Rehabilitation*.

[4] Lewington S, Whitlock G, Clarke R (2007). Blood cholesterol and vascular mortality by age, sex, and blood pressure: a meta-analysis of individual data from 61 prospective studies with 55,000 vascular deaths. *The Lancet*.

[5] Gordon T, Castelli WP, Hjortland MC, Kannel WB, Dawber TR (1977). High density lipoprotein as a protective factor against coronary heart disease. The Framingham Study. *The American Journal of Medicine*.

[6] Barter P, Gotto AM, LaRosa JC (2007). HDL cholesterol, very low levels of LDL cholesterol, and cardiovascular events. *The New England Journal of Medicine*.

[7] Clarke R, Emberson JR, Parish S (2007). Cholesterol fractions and apolipoproteins as risk factors for heart disease mortality in older men. *Archives of Internal Medicine*.

[8] Abbott RD, Wilson PW, Kannel WB, Castelli WP (1988). High density lipoprotein cholesterol, total cholesterol screening, and myocardial infarction. The Framingham study. *Arteriosclerosis*.

[9] Zimetbaum P, Frishman WH, Ooi WL (1992). Plasma lipids and lipoproteins and the incidence of cardiovascular disease in the very elderly. The Bronx Aging Study. *Arteriosclerosis and Thrombosis*.

[10] Florvall G, Basu S, Larsson A (2006). Apolipoprotein A1 is a stronger prognostic marker than are HDL and LDL cholesterol for cardiovascular disease and mortality in elderly men. *The Journals of Gerontology Series A*.

[11] Arai Y, Hirose N (2004). Aging and HDL metabolism in elderly people more than 100 years old. *Journal of Atherosclerosis and Thrombosis*.

[12] Hedberg P, Lönnberg I, Jonason T, Nilsson G, Pehrsson K, Ringqvist I (2001). Left ventricular systolic dysfunction in 75-year-old men and women. A population-based study. *European Heart Journal*.

[13] NCEP (2001). *Third report of the National Cholesterol Education Program (NCEP) Expert Panel on Detection, Evaluation, and Treatment of High Blood Cholesterol in Adults (adult treatment panel III)*.

[14] Grundy SM, Balady GJ, Criqui MH (1998). Primary prevention of coronary heart disease: guidance from Framingham: a statement for healthcare professionals from the AHA Task Force on Risk Reduction. American Heart Association. *Circulation*.

[15] Sharrett AR, Ballantyne CM, Coady SA (2001). Coronary heart disease prediction from lipoprotein cholesterol levels, triglycerides, lipoprotein(a), apolipoproteins A-I and B, and HDL density subfractions: the Atherosclerosis Risk in Communities (ARIC) Study. *Circulation*.

[16] Ingelsson E, Schaefer EJ, Contois JH (2007). Clinical utility of different lipid measures for prediction of coronary heart disease in men and women. *The Journal of the American Medical Association*.

[17] Wang J, Ruotsalainen S, Moilanen L, Lepistö P, Laakso M, Kuusisto J (2007). The metabolic syndrome predicts cardiovascular mortality: a 13-year follow-up study in elderly non-diabetic Finns. *European Heart Journal*.

[18] Shai I, Rimm EB, Hankinson SE (2004). Multivariate assessment of lipid parameters as predictors of coronary heart disease among postmenopausal women: potential implications for clinical guidelines. *Circulation*.

[19] Toth PP (2005). High-density lipoprotein as a therapeutic target: clinical evidence and treatment strategies. *The American Journal of Cardiology*.

[20] Manolio TA, Cushman M, Gottdiener JS, Dobs A, Kuller LH, Kronmal RA (2004). Predictors of falling cholesterol levels in older adults: the Cardiovascular Health Study. *Annals of Epidemiology*.

[21] Weverling-Rijnsburger AWE, Blauw GJ, Lagaay AM, Knook DL, Meinders AE, Westendorp RGJ (1997). Total cholesterol and risk of mortality in the oldest old. *The Lancet*.

[22] Neaton JD, Blackburn H, Jacobs D (1992). Serum cholesterol level and mortality findings for men screened in the Multiple Risk Factor Intervention Trial. *Archives of Internal Medicine*.

[23] Neaton JD, Wentworth D (1992). Serum cholesterol, blood pressure, cigarette smoking, and death from coronary heart disease: overall findings and differences by age for 316 099 white men. *Archives of Internal Medicine*.

[24] Smith GD, Shipley MJ, Marmot MG, Rose G (1992). Plasma cholesterol concentration and mortality: the Whitehall Study. *The Journal of the American Medical Association*.

[25] Expert Panel on Detection, Evaluation and Treatment of High Blood Cholesterol in Adults (2001). Executive summary of the third report of the National Cholesterol Education Program (NCEP) expert panel on detection, evaluation, and treatment of high blood cholesterol in adults (adult treatment panel III). *The Journal of the American Medical Association*.

[26] Krumholz HM, Seeman TE, Merrill SS (1994). Lack of association between cholesterol and coronary heart disease mortality and morbidity and all-cause mortality in persons older than 70 years. *The Journal of the American Medical Association*.

[27] Anum EA, Adera T (2004). Hypercholesterolemia and coronary heart disease in the elderly: a meta-analysis. *Annals of Epidemiology*.

[28] Benfante R, Reed D (1990). Is elevated serum cholesterol level a risk factor for coronary heart disease in the elderly?. *The Journal of the American Medical Association*.

[29] Schatz IJ, Masaki K, Yano K, Chen R, Rodriguez BL, Curb JD (2001). Cholesterol and all-cause mortality in elderly people from the Honolulu Heart Program: a cohort study. *The Lancet*.

[30] Kronmal RA, Cain KC, Ye Z, Omenn GS (1993). Total serum cholesterol levels and mortality risk as a function of age: a report based on the Framingham data. *Archives of Internal Medicine*.

[31] (2005). JBS 2: Joint British Societies' guidelines on prevention of cardiovascular disease in clinical practice. *Heart*.

[32] (1994). Randomised trial of cholesterol lowering in 4444 patients with coronary heart disease: the Scandinavian Simvastatin Survival Study (4S). *The Lancet*.

[33] Byington RP, Jukema JW, Salonen JT (1995). Reduction in cardiovascular events during pravastatin therapy: pooled analysis of clinical events of the 
Pravastatin Atherosclerosis Intervention Program. *Circulation*.

[34] LaRosa JC, He J, Vupputuri S (1999). Effect of statins on risk of coronary disease. A meta-analysis of randomized controlled trials. *The Journal of the American Medical Association*.

[35] Baigent C, Keech A, Kearney PM (2005). Efficacy and safety of cholesterol-lowering treatment: prospective meta-analysis of data from 90,056 participants in 14 randomised trials of statins. *The Lancet*.

[36] Heart Protection Study Collaborative Group (2002). MRC/BHF Heart Protection Study of cholesterol lowering with simvastatin in 20 536 high-risk individuals: a randomised placebo-controlled trial. *The Lancet*.

[37] Downs JR, Clearfield M, Weis S (1998). Primary prevention of acute coronary events with lovastatin in men and women with average cholesterol levels: results of AFCAPS/TexCAPS. Air Force/Texas Coronary Atherosclerosis Prevention Study. *The Journal of the American Medical Association*.

